# Regional Variation in Genetic Control of Atherosclerosis in Hyperlipidemic Mice

**DOI:** 10.1534/g3.120.401856

**Published:** 2020-10-27

**Authors:** Michael B. Jones, Alexander An, Lisa J. Shi, Weibin Shi

**Affiliations:** * Departments of Radiology & Medical Imaging,; † Biochemistry & Molecular Genetics, University of Virginia, Charlottesville, Virginia

**Keywords:** Atherosclerosis, Carotid artery, Aorta, Genetic loci, Site Specificity

## Abstract

Atherosclerosis is a polygenic disorder that often affects multiple arteries. Carotid arteries are common sites for evaluating subclinical atherosclerosis, and aortic root is the standard site for quantifying atherosclerosis in mice. We compared genetic control of atherosclerosis between the two sites in the same cohort derived from two phenotypically divergent *Apoe*-null (*Apoe*^−/−^) mouse strains. Female F2 mice were generated from C57BL/6 (B6) and C3H/He (C3H) *Apoe*^−/−^ mice and fed 12 weeks of Western diet. Atherosclerotic lesions in carotid bifurcation and aortic root and plasma levels of fasting lipids and glucose were measured. 153 genetic markers across the genome were typed. All F2 mice developed aortic atherosclerosis, while 1/5 formed no or little carotid lesions. Genome-wide scans revealed 3 significant loci on chromosome (Chr) 1, Chr15, 6 suggestive loci for aortic atherosclerosis, 2 significant loci on Chr6, Chr12, and 6 suggestive loci for carotid atherosclerosis. Only 2 loci for aortic lesions showed colocalization with loci for carotid lesions. Carotid lesion sizes were moderately correlated with aortic lesion sizes (*r* = 0.303; *P* = 4.6E-6), but they showed slight or no association with plasma HDL, non-HDL cholesterol, triglyceride, or glucose levels among F2 mice. Bioinformatics analyses prioritized *Cryge* as a likely causal gene for *Ath30*, *Cdh6* and *Dnah5* as causal genes for *Ath22*. Our data demonstrate vascular site-specific effects of genetic factors on atherosclerosis in the same animals and highlight the need to extend studies of atherosclerosis to sites beyond aortas of mice.

Atherosclerosis is a complex chronic inflammatory disease that often affects multiple large and medium arteries, specifically the aorta, coronary arteries, iliofemoral arteries, and the carotid bifurcations (Bentzon *et al.* 2014). Plague enlargement and rupture in the coronary arteries and carotid arteries are associated with major adverse clinical events, such as heart attack and ischemic stroke (Arbab-Zadeh *et al.* 2012). Heart attack and stroke are leading causes of death and disability in the U.S (Virani *et al.* 2020) and worldwide (https://www.who.int/health-topics/cardiovascular-diseases/).

Atherosclerotic artery disease has a strong genetic component. Limited information suggests that the effect of genetic factors on atherosclerosis development varies between vascular sites. Peripheral vascular disease is prominent in patients with type III hyperlipoproteinemia, a genetic disorder due to defective APOE2 and featured by elevated VLDL (Feussner *et al.* 1993), while coronary arterial disease tends to be severe in individuals with Tangier disease caused by ABCA1 deficiency (Schaefer *et al.* 2010). Of the 11 significant loci thus far identified by genome-wide association studies for carotid artery intima thickness, a measure of subclinical carotid atherosclerosis in humans (Franceschini *et al.* 2018)(Bis *et al.* 2011), 10 show no associations with coronary heart disease (Erdmann *et al.* 2018). In mice, quantitative trait loci (QTL) identified for atherosclerotic lesions at the aortic arch are distinct from those for lesions in the aortic root of F2 mice derived from C57BL6 (B6) and 129 *Apoe*-null (*Apoe*^−/−^) mice (Tomita *et al.* 2010). As atherosclerosis in the carotid arteries is a major cause of ischemic stroke (Lovett *et al.* 2004)^,^(Flaherty *et al.* 2013), we have applied the *Apoe*^−/−^ mouse model to the identification of genetic factors contributing to carotid plaque formation. Among the 8 significant loci identified for carotid atherosclerosis in mice (Li *et al.* 2008)(Rowlan *et al.* 2013a) (Grainger *et al.* 2017), half have no corresponding loci for aortic atherosclerosis. Atherosclerosis development is heavily influenced by environmental factors, which vary from study to study. An effective strategy to minimize environmental impact on vascular site-specific disparities in atherosclerosis is to examine plaque formation at different anatomic sites in the same animals. However, no study has been performed with the same animals to compare genetic influences on atherosclerosis development between aortas and carotid arteries.

B6 and C3H are two mouse strains that exhibit marked differences in atherosclerotic lesion formation at both the aortic root and the carotid arteries. C3H mice develop no or much smaller atherosclerotic lesions in aortas than B6 mice when fed an atherogenic diet or deficient in apolipoprotein E (Paigen *et al.* 1990)^,^(Shi *et al.* 2000). On a Western diet, B6-*Apoe*^−/−^ mice develop advanced plaques in the carotid arteries, but C3H-*Apoe*^−/−^ mice form no lesions (Zhao *et al.* 2019). The marked disparity between the two strains at both vascular sites in plaque formation offers an opportunity to understand regional variation in genetic control of atherosclerosis. Thus, in the present study, we performed genetic analysis to identify genetic loci affecting atherosclerosis development at the aortic root and the common carotid bifurcation in the same F2 cohort derived from the two *Apoe*^−/−^ strains.

## Materials and Methods

### Mice

A female F2 cohort was generated from an intercross between B6-*Apoe*^−/−^ and C3H-*Apoe*^−/−^ mice as reported (Li *et al.* 2008). Briefly, female B6-*Apoe*^−/−^ mice were crossed with male C3H-*Apoe*^−/−^ mice to generate F1 hybrids, which were intercrossed by brother–sister mating to generate 241 female F2 mice. Mice were started on a Western diet containing 21% fat, 34.1% sucrose, 0.15% cholesterol, and 19.5% casein (Evigo, TD 88137) at 6 weeks of age and maintained on the diet for 12 weeks. All procedures were carried out in accordance with the current National Institutes of Health guidelines and approved by the Institutional Animal Care and Use Committee.

### Measurement of atherosclerotic lesions

Atherosclerotic lesions in the aortic root and the left common carotid bifurcation were quantified as described previously (Li *et al.* 2008)^,^(Su *et al.* 2006a)^,^(Zhang *et al.* 2012). Briefly, after mice were killed by isoflurane inhalation, the vasculature was perfusion-fixed with 10% formalin through the heart. The distal portion of the left common carotid artery and its adjacent branches were harvested and embedded in OCT compound (Tissue-Tek). Serial 10-μm-thick cryosections were collected every 3 sections. The aortic root and adjacent portion of the heart were dissected, embedded in OCT compound, and cross-sectioned in 10-µm thickness, as reported (Su *et al.* 2006a). Sections were stained with oil red O and hematoxylin and counterstained with light green. Lesion sizes were measured using an ocular with a square-micrometer grid on a light microscope.

### Measurements of plasma lipids and glucose

Blood samples were collected from the retro-orbital venous plexus of mice after being fasted overnight with the animals under isoflurane anesthesia. Plasma levels of total cholesterol, high-density lipoprotein (HDL) cholesterol, and triglyceride were measured with Thermo DMA assay kits (Louisville, CO), as reported (Tian *et al.* 2005). Non-HDL cholesterol concentrations were calculated as the difference between total and HDL cholesterol levels. Plasma glucose concentrations were determined with a Sigma assay kit (Cat. # GAHK20). Briefly, 6 μl of diluted plasma (3x dilution in distilled water), together with standards and controls, were loaded in a 96-well plate and then mixed with 150 µl of assay reagent in each well. After a 30-min incubation at 30°, the absorbance at 340 nm was read on a Molecular Devices (Menlo Park, CA, USA) plate reader.

### Genotyping

Genomic DNA was isolated from cut tails and genotyped as described (Li *et al.* 2008). A total of 153 microsatellite markers across the entire genome at an average interval of 10 cM were typed.

### Statistical analysis

QTL analysis was performed using R/qtl and Map Manager QTX as previously reported (Fuller *et al.* 2020),(Grainger *et al.* 2017) (Garrett *et al.* 2017). One thousand permutations were run to define the genome-wide LOD (logarithm of odds) score threshold for significant or suggestive linkage to a particular trait. Loci that exceeded the LOD score threshold of 0.05 were considered significant (*P* < 0.05) and those exceeding the threshold of 0.63 were suggestive (*P* < 0.63). Regression analysis was performed to examine the relationship between two variables for F2 mice.

#### Prioritization of candidate genes:

When a significant QTL for atherosclerotic lesions was mapped in two or more crosses derived from different parental strains whose genome sequences were available, bioinformatics tools were used to prioritize positional candidate genes. Probable candidate genes were those that contained one or more nonsynonymous SNPs or a SNP in the upstream regulatory region and those SNPs were shared by the parental strains carrying the high allele but were different from the ones shared by the parental strains carrying the low allele at a QTL, as reported (Grainger *et al.* 2016)^,^(Rowlan *et al.* 2013b). SNPs, indels and structure variations (SVs) were queried via the Sanger Mouse Genomes Project database (https://www.sanger.ac.uk/sanger/Mouse_SnpViewer/rel-1505). SIFT (Sorting Intolerant From Tolerant) score was obtained through the Ensembl Genome Browser (https://useast.ensembl.org/index.html) and used for estimating the impact of an amino acid substitution on protein function (Vaser *et al.* 2016). We used available eQTL for mouse atherosclerotic lesions to prioritize positional candidate genes that contain one or more SNPs in intronic, 5′- and 3′-UTR regions for significant QTL (Bennett *et al.* 2015).

### Data availability

All data used in this article are included in Supplemental Materials. Supplemental material available at figshare: https://doi.org/10.25387/g3.13146539.

## Results

### Penetrance of carotid atherosclerosis *vs.* aortic atherosclerosis

**Figure 1:**
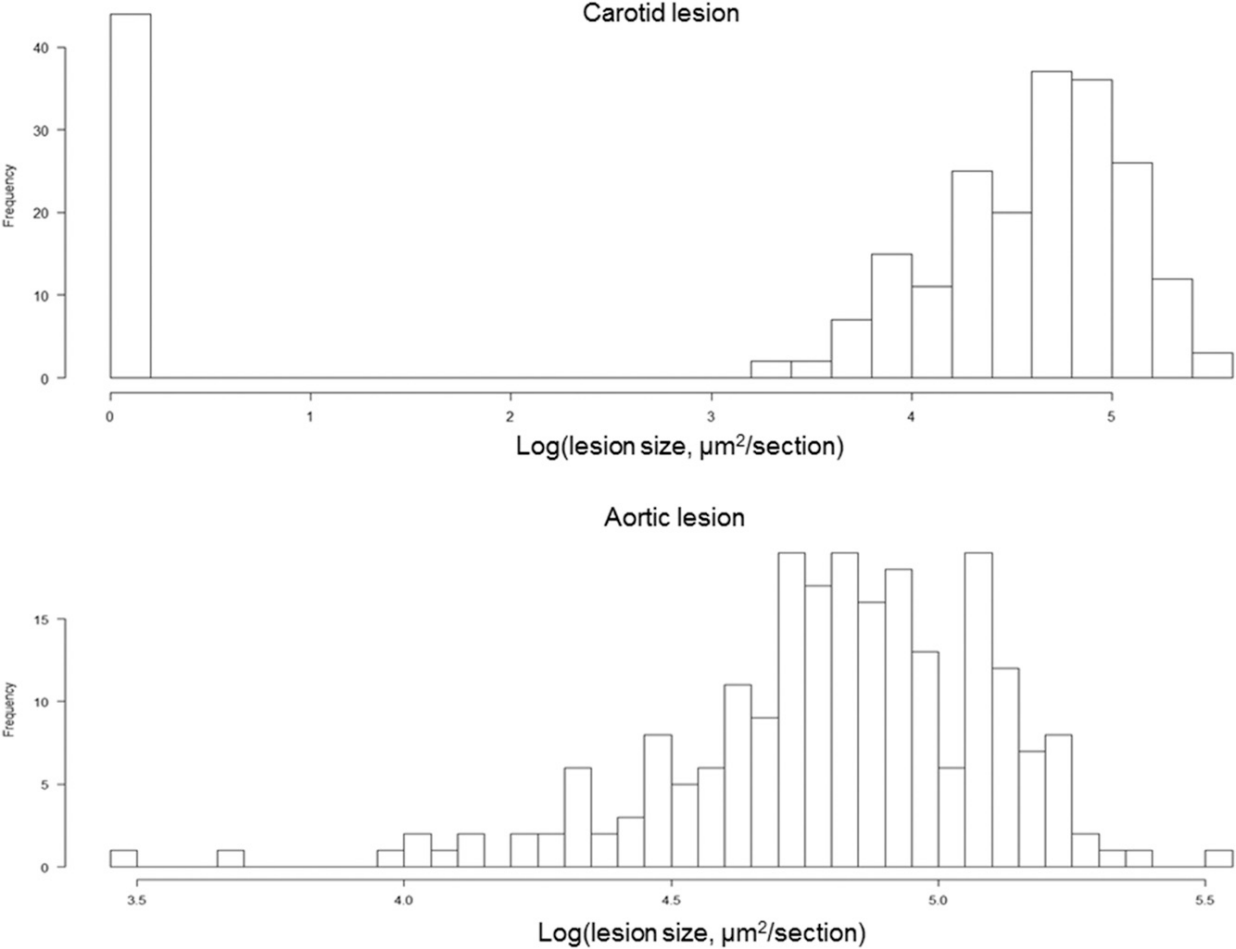
Distributions of log-transformed atherosclerotic lesion sizes in the carotid artery (top) and log-transformed atherosclerotic lesion sizes in the aortic root (bottom) of female F2 mice derived from B6-*Apoe*^−/−^ and C3H-*Apoe*^−/−^ mice. F2 mice were fed 12 weeks of Western diet. The graphs were created with a plot function of R/qtl.

After being fed 12 weeks of Western diet, all F2 mice developed atherosclerotic lesions at the aortic root, while 44 of the 241 F2 mice (18.3%) formed no lesions in the carotid artery (Figure 1). The values of log-transformed aortic lesion sizes of F2 mice were approximately normally distributed. In contrast, the values of log-transformed carotid lesion sizes of F2 mice exhibited a bimodal distribution: The rectangle bar on the left edge represents F2 mice that had no lesion, and the bell-shaped histogram on the right represents mice with various sizes of carotid lesions.

### Genetic loci for aortic atherosclerosis *vs.* carotid atherosclerosis

**Figure 2:**
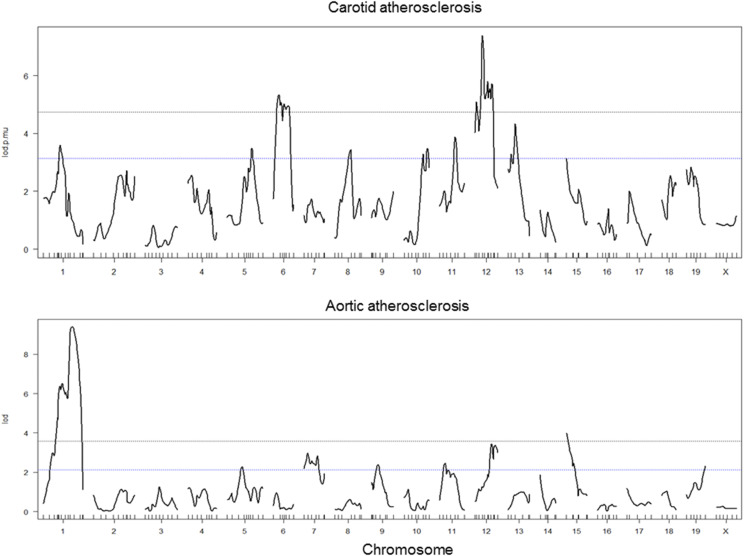
Genome-wide scans to search for loci affecting carotid and aortic atherosclerotic lesion sizes of F2 mice. Chromosomes 1 through 19 and X are represented numerically on the X-axis. Each short vertical bar on the X-axis represents a genetic marker. The relative width of the space between two short vertical bars reflects the relative length on a chromosome. The Y-axis represents the LOD (logarithm of odds) score. Two horizontal dot lines denote genome-wide thresholds for suggestive (*P* = 0.63) and significant (*P* = 0.05) linkage.

Whole genome-wide scans were performed on F2 mice to search for loci affecting atherosclerotic lesion sizes in the aortic root and the left carotid bifurcation. 3 significant QTL on Chr1 and Chr15 and 6 suggestive QTL on Chr5, 7, 9, 11, 12, and 19 were identified for aortic lesions (Figure 2). 2 significant QTL on Chr6 and Chr12 and 6 suggestive QTL on Chr1, 5, 8, 10, 11, and 13 were found for carotid lesions. Details of these QTL, including locus name, LOD score, peak location, 95% confidence interval (CI), high allele, mode of inheritance, and allelic effect are presented in Table 1.

**Table 1:** Significant and suggestive QTL for aortic and carotid atherosclerosis mapped in F2 mice derived from B6 and C3H Apoe^−/−^ mice

Locus name	Chr	LOD*^a^*	Peak (cM)	Closest marker	95%CI^b^	High allele	Mode of inheritance^c^	Allelic effect^d^		
								BB	BC	CC
*Cath10*	1	3.59	43.7	D1Mit45	32.7-57.7	B	Additive	17.7 ± 18.0	14.2 ± 14.9	8.4 ± 10.1
*Cath2*	5	3.48	61.4	D5Mit95	39.4-71.4	C	Dominant	9.8 ± 14.2	15.2 ± 15.9	13.8 ± 13.9
***Cath4***	6	**5.33**	28.1	D6Mit243	20.1-56.1	B	Additive	17.5 ± 18.2	13.6 ± 13.8	7.7 ± 10.7
*Cath11*	8	3.42	50.7	D8Mit211	32.7-55.7	B	Dominant	13.5 ± 13.0	14.5 ± 17.0	11.2 ± 12.9
*Cath12*	10	3.48	66.0	D10Mit103	49-70	B	Dominant	14.6 ± 14.5	14.7 ± 16.2	9.0 ± 10.9
*Cath9*	11	3.88	47.9	D11Mit36	40.9-54.9	B	Additive	17.8 ± 17.0	13.7 ± 14.7	7.5 ± 10.4
***Cath1***	12	**7.39**	24.0	D12Mit285	21-29	B	Additive	21.1 ± 19.0	13.0 ± 13.2	6.5 ± 8.3
*Cath3*	13	4.33	30.1	D13Mit250	17.9-38.9	B	Additive	18.1 ± 15.2	13.1 ± 15.3	8.9 ± 11.7
***Ath30***	1	**6.2**	46.7	D1Mit45	39.9-55.8	B	Additive	97.2 ± 46.1	79.7 ± 45.8	57.3 ± 37.7
***Ath1***	1	**9.40**	72.67	D1Mit425	65.7-86.7	B	Additive	105.6 ± 55.3	75.1 ± 37.8	48.3 ± 26.7
*Ath42*	5	2.27	37.35	D5Mit309	9.4-86.6	B	Additive	89.9 ± 55.6	76.8 ± 41.4	65.8 ± 45.8
*Ath31*	7	2.97	25.61	D7Mit228	17.1-65.2	B	Additive	95.5 ± 50.0	80.3 ± 48.1	60.6 ± 31.3
*Ath29*	9	2.38	32.80	D9Mit260	17.8-52.8	B	Additive	87.5 ± 46.2	80.2 ± 48.3	58.7 ± 32.2
*Ath46*	11	2.45	23.90	D11Mit236	12.9-56.9	B	Recessive	90.4 ± 43.8	72.0 ± 46.9	75.1 ± 43.8
*Ath21*	12	3.44	46.00	D12Mit239	39.0-60.6	B	Dominant	83.6 ± 43.7	83.4 ± 45.6	59.4 ± 36.7
***Ath22***	15	**3.98**	3.82	D15Mit80	3.8-19.6	C	Dominant	61.0 ± 50.9	83.9 ± 44.0	80.0 ± 41.6
*Ath16*	19	2.31	48.46	D19Mit103	3.4-48.5	—	Heterosis	74.3 ± 39.5	86.3 ± 49.9	63.4 ± 39.6

a LOD scores were obtained from genome-wide QTL analysis using R/qtl. Significant QTL and LOD score were highlighted in bold. The suggestive and significant LOD score thresholds were defined by 1,000 permutation tests for the traits. Suggestive and significant LOD score thresholds for carotid atherosclerosis were 3.14 and 4.74, respectively. Suggestive and significant LOD score thresholds for aortic atherosclerosis were 2.13 and 3.58, respectively. ^b^ 95% Confidence interval in cM was estimated as the interval under the LOD curve after a 1.8-LOD unit drop from the peak. ^c^ Mode of inheritance was defined based on allelic effect of the nearest marker at a QTL. C: C3H allele; B: B6 allele. ^d^ Measurements for atherosclerosis are expressed as means ± SD in lesion size (µm^2^/section x 1000).

**Figure 3:**
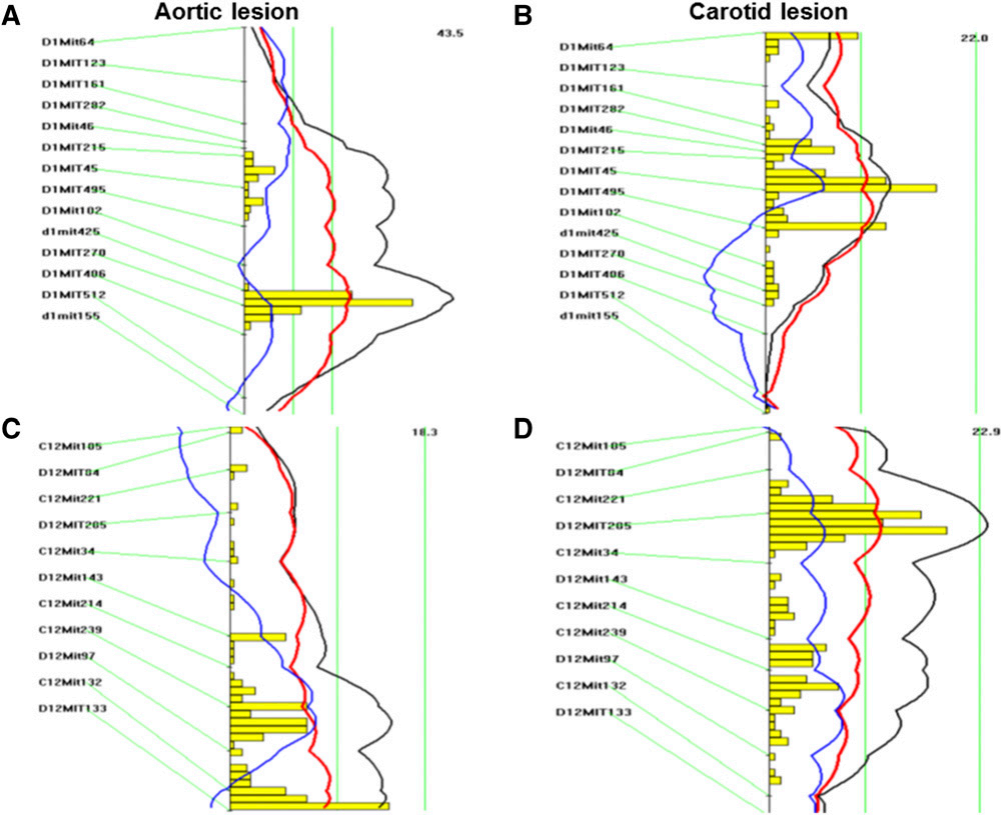

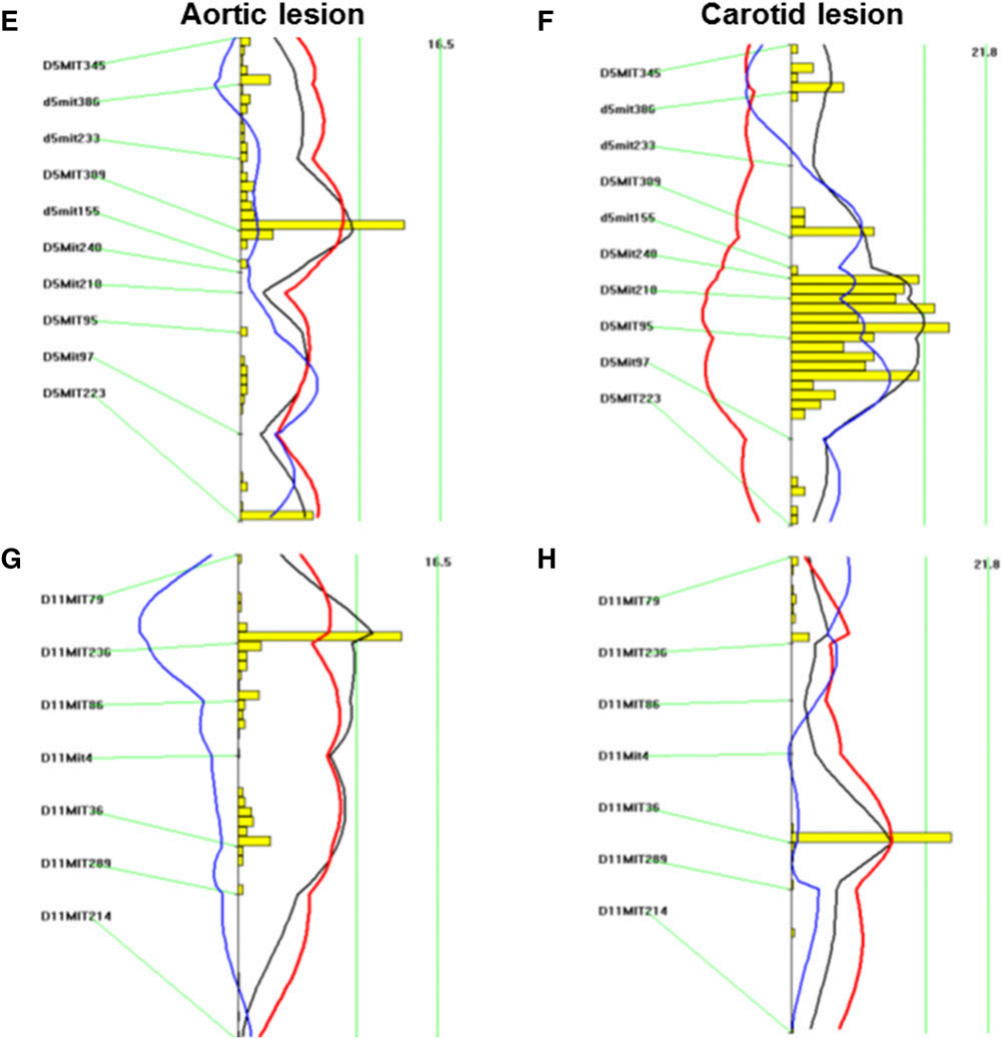
Interval mapping graphs for aortic and carotid atherosclerotic lesion sizes on chromosomes 1 (A, B), 12 (C, D), 5 (E, F), and 11 (G, H) where QTL for both traits were detected. Plots were made with the interval mapping function of Map Manager QTX. Yellow histograms estimate the confidence intervals for detected QTL. Two green vertical lines denote genome-wide significance thresholds for suggestive or significant linkage (*P* = 0.63 and *P* = 0.05, respectively). The black curved line represents the LOD score calculated at 1-cM intervals, the red and blue lines denote additive and dominance effects, respectively.

Interval mapping graph for Chr1 showed 2 adjacent QTL affecting aortic atherosclerosis (Figure 3A). The distal QTL had a highly significant LOD score of 9.4 and peaked at 72.67 cM. This QTL replicates *Ath1*, mapped initially in recombinant inbred strains derived from B6 and BALB/c and also from B6 and C3H mice and replicated in multiple intercrosses (Grainger *et al.* 2016) (Zhang *et al.* 2012)^,^(Rowlan *et al.* 2013b)^,^ (Wang *et al.* 2007). The proximal QTL had a highly significant LOD score of 6.2 and peaked at 46.7 cM. This QTL replicates *Ath30*, mapped previously in two B x H crosses and a 129 x DBA/2 F2 cross (Wang *et al.* 2007)^,^(Rowlan *et al.* 2013b)^,^(Kayashima *et al.* 2014). It overlaps with a locus for carotid atherosclerosis, *Cath10*, which peaked at 43.7 cM and had a LOD score of 3.59 (Figure 3B).

The QTL on Chr15 for aortic atherosclerosis had a significant LOD score of 3.98 and peaked at 3.82 cM (Table 1). This QTL replicates *Ath22*, previously mapped in a DBA/2 x AKR cross (Smith *et al.* 2006). Unlike other QTL for aortic atherosclerosis where the B6 allele was associated with an increased lesion size and the C3H allele associated with a decreased lesion size, this QTL derived its high allele from C3H and low allele from B6 mice (Table 1).

Interval mapping graph for Chr12 showed the existence of 2 QTL affecting carotid atherosclerosis: The proximal one peaked at 24 cM near *D12Mit285* and the distal one peaked at 37.86 cM near *D12Mit214* (Figure 3D). The proximal QTL, *Cath1*, was previously mapped in B6 x BALB/c and BALB/c x SM intercrosses (Grainger *et al.* 2017) (Rowlan *et al.* 2013a). The distal QTL overlaps with a suggestive QTL for aortic atherosclerosis, which peaked at 46 cM (Figure 3C). A significant QTL on Chr6 was identified for carotid atherosclerosis, which replicates *Cath4* previously mapped in a B6 x BALB/c cross (Rowlan *et al.* 2013a).

A suggestive QTL for carotid atherosclerosis on Chr5 was partially overlapping with a suggestive QTL for aortic atherosclerosis in the confidence interval, but they peaked at a different location (Figure 3E, 3F). The QTL for carotid atherosclerosis peaked at 61.4 cM, which replicates *Cath2* previously mapped in a B6 x BALB/c intercross (Rowlan *et al.* 2013a), while the QTL for aortic atherosclerosis peaked at 37.35 cM, replicating *Ath42* (Zhang *et al.* 2012). Allelic effects of these two QTL were different in that the C3H allele was associated with increased carotid atherosclerosis but decreased aortic atherosclerosis (Table 1).

A suggestive QTL on Chr11 was also identified for both carotid and aortic atherosclerosis, but they occurred at a different chromosomal region (Figure 3G, 3H). The QTL for carotid atherosclerosis, *Cath10*, peaked at 47.9 cM, while the QTL for aortic atherosclerosis peaked at 23.9 cM (Table 1). The latter replicates a suggestive QTL for aortic lesions mapped previously in a B x H intercross (Su *et al.* 2006a); thus named *Ath46*. The mode of inheritance was also different in that the B6 allele increased carotid atherosclerosis in an additive mode but increased aortic atherosclerosis in a recessive mode.

The QTL on Chr8, 10, and 13 for carotid atherosclerosis did not overlap with any QTL for aortic atherosclerosis. The QTL on Chr13 had a LOD score of 4.33 and peaked at 30.1 cM (Table 1). This QTL overlapped with *Cath3*, previously mapped in the B6 x BALB/c intercross (Rowlan *et al.* 2013a). The QTL on Chr8 and Chr10 were novel. The former QTL, named *Cath11*, peaked at 50.7 cM and had a LOD score of 3.42. The latter QTL, named *Cath12*, peaked at 66 cM and had a LOD score of 3.48.

For aortic atherosclerosis, the suggestive QTL near 25.6 cM on Chr7 replicates *Ath31*, the QTL near 32.8 cM on Chr9 replicates *Ath29*, previously mapped in B x H intercrosses (Wang *et al.* 2007)^,^(Su *et al.* 2006a), the QTL at 46 cM on Chr12 overlaps with interacting locus *Ath21* identified in a B6 × 129S1 cross (Ishimori *et al.* 2004), and the QTL at 48.46 cM on Chr19 replicates *Ath16* mapped in a B6 x FVB/N cross (Dansky *et al.* 2002).

### Prioritization of candidate genes

The proximal QTL on Chr1 for aortic atherosclerosis, *Ath30*, was mapped in this cross and 3 previous crosses, including 2 B x H crosses and a 129 x DBA/2 F2 cross (Wang *et al.* 2007)^,^(Rowlan *et al.* 2013b)^,^(Kayashima *et al.* 2014). This QTL was also overlapping with *Cath10* for carotid atherosclerosis. At the QTL, B6 and DBA/2 alleles were associated with a larger lesion size while C3H and129 alleles were associated with a smaller lesion size. The Sanger SNP dataset was searched to find candidate genes that contain nonsynonymous SNP(s) or SNP(s) in upstream regulatory regions that are shared by the low allele strains (129 and C3H) but different from ones shared by the high allele strains (B6 and DBA/2) within the confidence interval (Table 2). *Cryge*, *Mogot*, *Utp14b*, *Scg2*, *Nyap2*, and *B3gnt7* contain one or more nonsynonymous SNPs, but only *Cryge* contains 2 SNPs that have a “0” SIFT score. Amino acid substitutions at 7 (E/G) and 94 (T/I) are predicted to have detrimental effects on protein function.

**Table 2:** Haplotype analysis for *Ath30* on chromosome 1 and *Ath22* on chromosome 15

Ath30 (50-90 Mb)				High allele		Low allele					
Chr	Position	Gene	dbSNP	B6	DBA_2J	129S1_SvIm	C3H_HeJ	Csq	Amino acid	AA coordinate	SIFT
1	64690848	Ccnyl1	rs3682263	C	—	G	G	5_prime_utr_variant			
1	64737532	Fzd5	rs30864875	A	—	G	G	5_prime_utr_variant			
**1**	**65050833**	**Cryge**	**rs31896846**	**G**	**-**	**A**	**A**	**missense_variant**	**T/I**	**94**	**0**
**1**	**65051094**	**Cryge**	**rs30805491**	**T**	**-**	**C**	**C**	**missense_variant**	**E/G**	**7**	**0**
1	78499361	BC035947	—	G	—	A	A	missense_variant	—	—	—
1	78499653	BC035947	—	G	—	A	A	missense_variant	—	—	—
1	78511226	Mogat1	—	A	—	G	G	splice_region_variant			
1	78537971	Mogat1	—	C	—	T	T	missense_variant	T/I	294	0.4
1	78665012	Utp14b	—	G	—	A	A	missense_variant	R/Q	209	1
1	79435347	Scg2	rs8253473	G	—	T	T	missense_variant	P/Q	553	1
1	79761838	Wdfy1	rs51597504	A	—	C	C	5_prime_utr_variant			
1	79776115	Mrpl44	rs50597413	T	—	C	C	5_prime_utr_variant			
1	81269388	Nyap2	rs30198875	A	—	G	G	missense_variant	—	—	—
1	85778602	A630001G21Rik	rs223873695	C	—	T	T	5_prime_utr_variant			
1	86303891	B3gnt7	rs31395357	C	—	T	T	5_prime_utr_variant			
1	86304255	B3gnt7	rs33048624	A	—	G	G	splice_region_variant			
1	86306217	B3gnt7	rs31277682	A	—	G	G	missense_variant	Q/R	395	0.09

Ath22 (1-30 Mb)				Low allele		High allele		Csq	Amino acid	AA coordinate	SIFT
Chr	Position	Gene	dbSNP	B6	AKR_J	C3H_HeJ	DBA_2J				
15	12818091	6030458C11Rik	rs3681822	C	—	T	T	missense_variant			
15	12818216	6030458C11Rik	rs32031213	C	—	T	T	missense_variant			
15	12821444	6030458C11Rik	rs31704434	A	—	G	G	missense_variant			
15	12824505	6030458C11Rik	rs48279007	T	—	G	G	missense_variant			
15	12824880	Drosha	rs38201751	C	—	G	G	5_prime_utr_variant			
15	12824925	Drosha	rs31833279	C	—	T	T	5_prime_utr_variant			
15	12833943	Drosha	rs13470543	G	—	A	A	missense_variant	Q	44	—
**15**	**13041377**	**Cdh6**	**rs31868071**	**T**	**-**	**C**	**C**	**missense_variant**	**S/G**	**534**	**0.03**
15	25148989	9230109A22Rik	rs32435878	A	—	G	G	5_prime_utr_variant			
15	25413714	Basp1	rs237680064	A	—	G	G	5_prime_utr_variant			
15	25414436	Gm5468	rs108432668	A	—	G	G	missense_variant			
15	27562809	Ank	rs31625299	C	—	T	T	missense_variant	A/V	201	0.37
15	28269390	Dnah5	rs256383837	T	—	C	C	missense_variant	V/A	892	0.61
15	28269407	Dnah5	rs32933300	G	—	T	T	missense_variant	A/S	898	0.79
15	28270500	Dnah5	rs32933295	C	—	T	T	missense_variant	H/Y	983	1
**15**	**28345815**	**Dnah5**	**rs31986690**	**C**	**-**	**T**	**T**	**missense_variant**	**R/W**	**2434**	**0.04**

Candidate genes with functional significance are denoted in bold. A low SIFT score hinting a high likelihood of changes in protein function is denoted in red. Chr, chromosome; dbSNP, Single Nucleotide Polymorphism Database identifier; SIFT, Sorting Intolerant From Tolerant; Csq, DNA sequence variation; UTR, untranslated region.

*Ath22* on Chr15 for aortic atherosclerosis was mapped in this cross and the previously reported AKR × DBA/2 *Apoe*^−/−^ cross (Smith *et al.* 2006). At the QTL, B6 and AKR alleles are the low alleles associated with smaller lesion sizes while C3H and DBA/2 alleles are the high allele associated with larger lesion sizes. We used the Sanger SNP dataset to prioritize *Drosha*, *Cdh6*, *Basp1*, *Ank*, and *Dnah5* as candidate genes containing nonsynonymous SNP(s) or SNP(s) in upstream regulatory regions that are shared by the low allele strains (B6 and AKR) but are different from SNP(s) shared by the high allele strain (C3H and DBA/2) in the confidence interval (Table 2). Of them, *Cdh6* and *Dnah5* contain a nonsynonymous SNP with a low SIFT score (0.03 and 0.04, respectively). The amino acid substitution at 534 (S/G) in the *Cdh6* protein and at 2,434 (R/W) in the *Dnah5* protein is predicted to impact protein function.

We also performed analysis by including all genetic variants, including those in intronic, 5′- and 3′-UTR regions, that were segregating between low allele strains and high allele strains, to prioritize candidate genes for *Ath30* and *Ath20* (Supplementary data). These genes were then evaluated for associations with atherosclerosis using the gene expression data for the aorta and liver of over 100 inbred mouse strains from the Hybrid Mouse Diversity Panel (HMDP) (Bennett *et al.* 2015). Of all candidate genes for *Ath30*, *Crygc* and *Mogat1*showed a negative correlation with atherosclerosis in their hepatic transcript levels, *Cyp27a1* showed a negative and *Acsl3* a positive correlation with atherosclerosis in aortic transcript levels. No candidate gene for *Ath22* showed any correlation with atherosclerosis in either aortic or hepatic transcript level.

### Correlation between carotid atherosclerosis and aortic atherosclerosis

**Figure 4:**
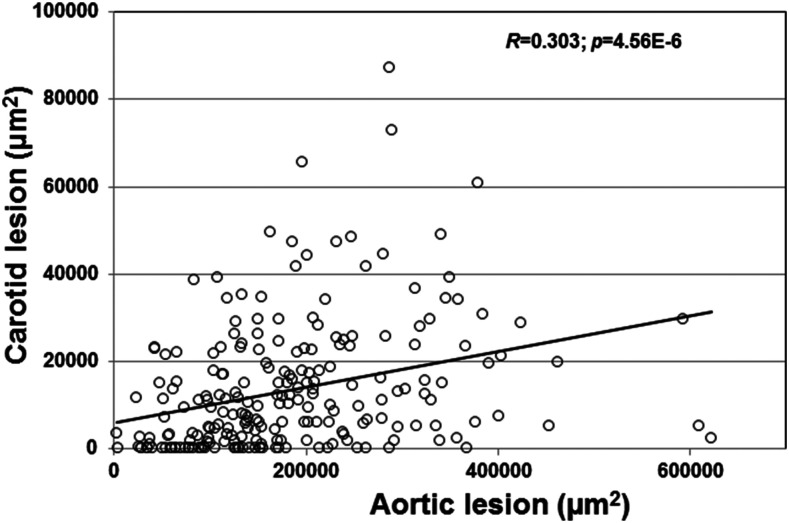
Correlation between aortic and carotid atherosclerotic lesion sizes of F2 mice. Each circle represents values of an individual F2 mouse. The *r*^2^ and *p* values are shown in the figure.

A moderate correlation between carotid lesion sizes and aortic lesion sizes was observed in the F2 mice (***r* = 0.303; *P* = 4.56E-6) (**Figure 4**).** F2 mice with larger aortic atherosclerosis tended to have larger carotid lesions, and vice versa.

### Associations of atherosclerotic lesions with plasma lipid and glucose levels

**Figure 5:**
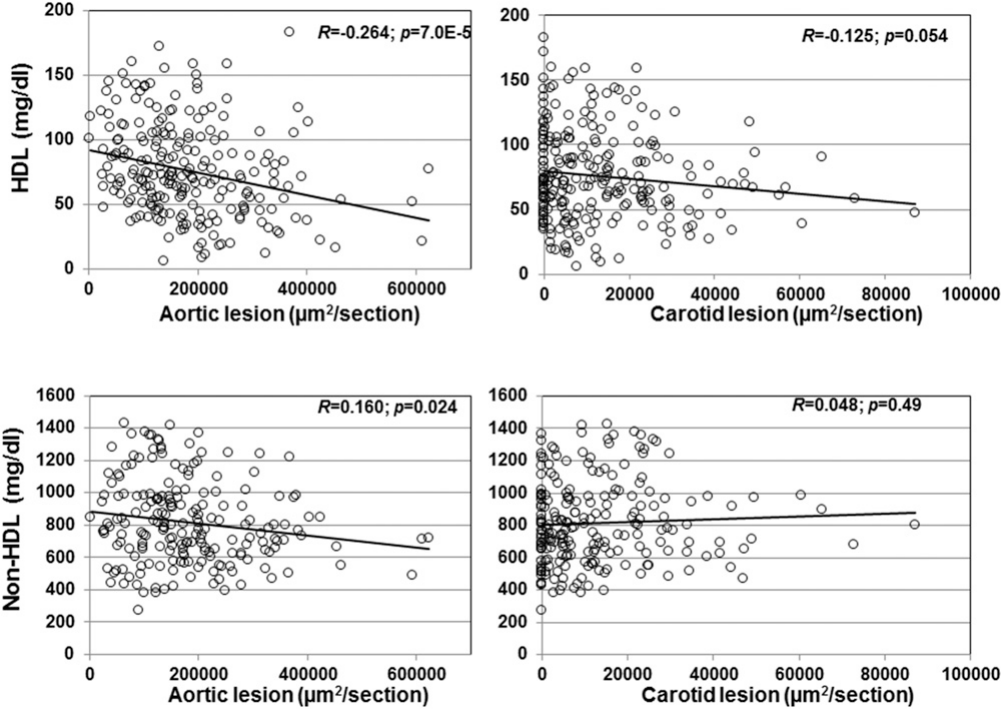

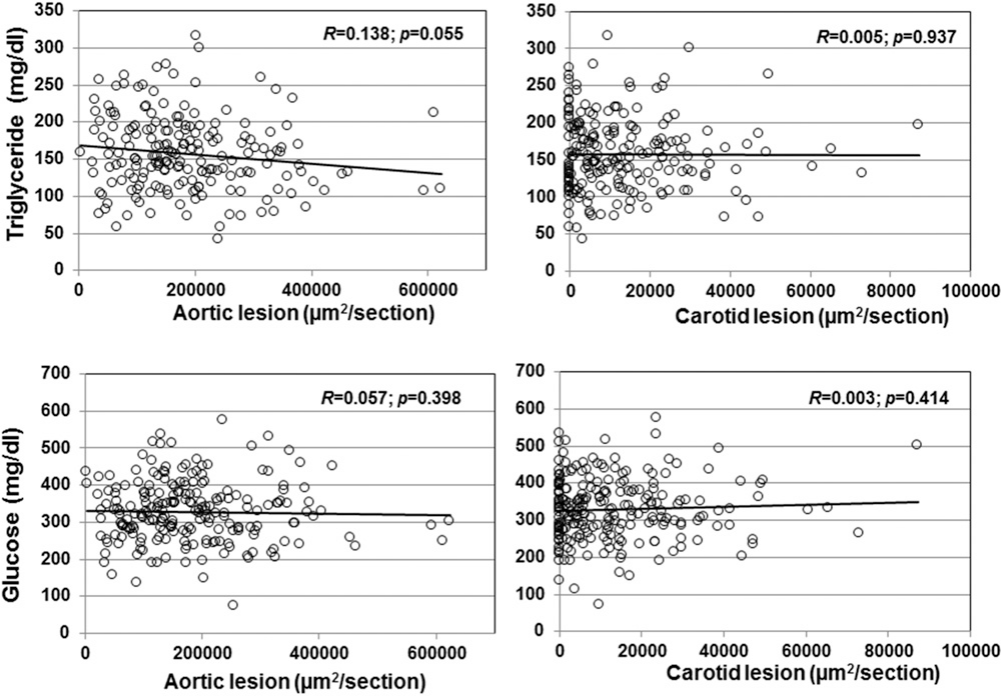
Correlations of aortic or carotid atherosclerotic lesion sizes with plasma levels of HDL, non-HDL cholesterol, triglyceride, and glucose in F2 mice fed the Western diet. Each circle represents values of an individual F2 mouse. The *r* and *p* values are shown in the figures.

A significant inverse correlation between aortic lesion sizes and HDL cholesterol levels was observed in the F2 cohort on the Western diet (r**=-0.264; *P* = 7.0E-5;** Figure 5). Carotid lesion sizes showed a trend toward a significant inverse correlation with HDL levels (***r*=-0.125; *P* = 0.054).** A slight but statistically significant correlation was also observed between aortic lesion sizes and non-HDL cholesterol levels (***r*=-0.160; *P* = 0.024**), but there was no correlation between carotid lesion sizes and non-HDL cholesterol levels (***r* = 0.048; *P* = 0.49)**. Neither carotid nor aortic lesions showed a significant correlation with plasma triglyceride or glucose levels.

## Discussion

In this study, we demonstrated that aortic atherosclerosis and carotid atherosclerosis were controlled by distinct genetic loci in the female F2 cohort derived from an intercross between B6 and C3H *Apoe*^−/−^ mice. Carotid atherosclerosis was controlled by 2 significant QTL on Chr6 and Chr12 and 6 suggestive QTL on Chr1, 5, 8, 10, 11, and 13, while aortic atherosclerosis was affected by 3 significant QTL on Chr1 and Chr15 and 6 suggestive QTL on Chr5, 7, 9, 11, 12, and 19. Of these QTL for aortic atherosclerosis, only the proximal QTL on Chr1 (*Ath30*) and the suggestive QTL on Chr12 (*Ath21*) showed colocalization with QTL for carotid atherosclerosis, thus demonstrating the site specificity of genetic effects on atherosclerosis.

Atherosclerotic lesions in the aortic root and the carotid artery of F2 mice were measured after 12 weeks on the Western diet. Under this condition, all F2 mice developed atherosclerotic lesions in the aortic root, but 1/5 of them had no or little lesions in the carotid artery. The incomplete penetrance of carotid atherosclerosis has also been observed in other F2 crosses derived from *Apoe*^−/−^ mouse strains and treated with identical conditions (Grainger *et al.* 2017)(Rowlan *et al.* 2013a). As the two arterial sites were exposed to the same systemic risk factors like hyperlipidemia, local factors, specifically vascular geometry, hemodynamics, and arterial wall properties, should be responsible for the difference in penetrance. Indeed, a previous study showed that genetic factors modulating vascular geometry also affect atherosclerotic lesion sizes in the aortic arch of mice (Tomita *et al.* 2010).

Carotid atherosclerosis have been studied in 3 separate intercrosses, including B6 x C3H, B6 x BALB/c, and BALB/c x SM *Apoe*^−/−^ F2s, and 8 significant QTL have been found (Li *et al.* 2008)^,^(Grainger *et al.* 2017) (Rowlan *et al.* 2013a). Most of the QTL identified are distinct from those for aortic atherosclerosis. However, genetic influences on atherogenesis have not been compared between the aorta and the carotid artery in the same animals. This study represents the first demonstration of the genetic disparity of atherogenesis for the two vascular beds in the same animals.

The three significant QTL identified for aortic atherosclerosis replicate *Ath1*, *Ath30* on Chr1 and *Ath22* on Chr15 previously reported (Paigen *et al.* 1987)^,^(Wang *et al.* 2007)^,^(Smith *et al.* 2006)^,^(Rowlan *et al.* 2013b). *Ath1* has been mapped in multiple crosses and *Tnfsf4* identified as its causal gene (Wang *et al.* 2005). *Ath22* was mapped in this cross and a previously reported AKR x DBA/2 intercross (Smith *et al.* 2006). By perusing genes containing one or more variants that were shared by the high allele strains (C3H, DBA/2) but were different from ones shared by the low allele strains (B6, AKR), we prioritized *Cdh6* and *Dnah5* as likely candidate genes for *Ath22*. This analysis is based on the fact that 97% of the genetic variants between inbred mouse strains are ancestral and thus genetic polymorphisms shared among common inbred strains almost certainly underlie the QTL genes (Wiltshire *et al.* 2003). As a QTL is derived from changes in the function or the amount of a gene product, we have focused on genes that carry a nonsynonymous SNP or a SNP in upstream regulatory region segregating between the high allele and the low allele strains of the genetic crosses. The amino acid substitution at 534 (S/G) in *Cdh6* and at 2,434 (R/W) in *Dnah5* was predicted to impact protein function due to their low SIFT scores. Flint *et al.* (Flint *et al.* 2005) analyzed 20 QTL identified in the mouse, and found that causal allelic variations were located in the coding region sequence or upstream regulatory sequence. Similarly, of the 3 QTL genes we identified, 2 have SNPs in the upstream regulatory region and 1 has a nonsynonymous SNP (Manichaikul *et al.* 2011) (Li *et al.* 2012)(Lu *et al.* 2011). Moreover, all of the identified rodent QTL have large phenotypic effects (4% variation). SNPs in downstream regions or introns may contribute to a complex trait or disease, but their effect sizes are expected to be weaker.

*Ath30* has been mapped in multiple crosses derived from different mouse strains, including B6, C3H, 129, and DBA/2 (Wang *et al.* 2007)^,^(Rowlan *et al.* 2013b)^,^(Kayashima *et al.* 2014). Moreover, this QTL overlaps with *Cath10* for carotid atherosclerosis. Using bioinformatics resources, we identified *Cryge* as a likely candidate gene for *Ath30*. Two amino acid substitutions (E7G and T94I) have a detrimental effect on protein function based on the 0 SIFT score. *Cryge* encodes crystallin, gamma E, which is a major component of the lens but also shows expression in the vessels and other tissues. However, its extralenticular functions remain unknown.

As carotid intima‐media thickness can be accurately measured with ultrasound, it has been clinically used to predict cardiovascular disease. In this study, we found that carotid lesion sizes were only moderately associated with plaque sizes in the aortic root of F2 mice. This result provides partial explanation for the inconsistent results regarding the association between carotid intima‐media thickness and future cardiovascular events. Large clinical studies have shown increased risk of myocardial infarction with each incremental increase of carotid intima-media thickness (Chambless *et al.* 1997)^,^(O’Leary *et al.* 1992)^,^(Bots *et al.* 1997)^,^(Lorenz *et al.* 2006). However, other studies have found little improvement in cardiovascular event prediction after carotid intima‐media thickness is added to conventional risk prediction (Den Ruijter *et al.* 2012)^,^(Yeboah *et al.* 2012)^,^(Simon *et al.* 2010).

Dyslipidemia and hyperglycemia are well established risk factors for atherosclerosis. Here, a significant inverse correlation was observed between HDL cholesterol levels and aortic lesion sizes in the F2 population fed the Western diet. Similar observations have also been made in other F2 crosses (Rowlan *et al.* 2013b)^,^(Wang *et al.* 2007). However, HDL only showed a trend toward a significant association with carotid lesion sizes in this cross. Marginal inverse correlations of HDL with carotid lesion sizes have been observed in previous crosses (Grainger *et al.* 2017) (Rowlan *et al.* 2013a). Together, these findings support the concept that HDL protects against atherosclerosis by inhibiting plaque growth (Feig *et al.* 2016) though there are conflicting reports (Shah *et al.* 2013)^,^(Holmes *et al.* 2015). Non-HDL cholesterol and triglyceride levels showed little or no association with either aortic or carotid lesion sizes in this cross. These results are consistent with previous observations made in other mouse crosses (Su *et al.* 2006a)^,^(Rowlan *et al.* 2013b)(Garrett *et al.* 2017) (Grainger *et al.* 2017). On the Western diet, *Apoe*^−/−^ mouse strains, including B6 and C3H, develop type 2 diabetes with fasting plasma glucose levels exceeding 250 mg/dl (Su *et al.* 2006b)^,^(Liu *et al.* 2015). The average fasting glucose levels of F2 mice in this cross were approximately 300 mg/dl. Although diabetes is a major risk factor for atherosclerosis, fasting glucose levels showed no association with either aortic or carotid lesion sizes in the F2 mice. This finding is consistent with our previous observations with other mouse crosses (Garrett *et al.* 2017)^,^(Grainger *et al.* 2017) (Grainger *et al.* 2016). It is noteworthy that all the F2 mice were Apoe-deficient and had marked elevations in non-HDL cholesterol and glucose levels on the Western diet so the associations achieved might not be extrapolated to humans.

In conclusion, we have demonstrated the site-specificity of genetic influences on atherosclerosis and a moderate correlation between carotid and aortic lesion sizes using the *Apoe*^−/−^ mouse model that develops all phases of atherosclerotic lesions seen in humans. The findings offer experimental evidence in support of the AHA/ACC recommendation against the use of carotid intima‐media thickness for individual risk prediction in clinical practice (Lloyd-Jones *et al.* 2013). Our data also highlight the need to extend studies to sites beyond the aorta of mice and to genes regulating interactions of local arterial walls with hemodynamic force and other risk factors during the atherogenesis process.
